# Age-Related Effects of Physical Performance on Technical and Tactical Outcomes in Youth Soccer

**DOI:** 10.3390/sports13060162

**Published:** 2025-05-27

**Authors:** Patrik Hegedüs, Dániel Csala, János Tóth, János Tóth

**Affiliations:** 1 Department of Football, Hungarian University of Sports Sciences, Alkotás utca 44-48, 1123 Budapest, Hungary; 2 Department of Kinesiology, Hungarian University of Sports Sciences, Alkotás utca 44-48, 1123 Budapest, Hungary; 3 Sports Science Department, Ferencvárosi TC, Üllői út 129, 1091 Budapest, Hungary

**Keywords:** football, running performance, technical–tactical performance, correlation, youth athletes

## Abstract

This study explores how age influences the relationship between physical performance (PP) and technical–tactical parameters (TPs) in youth soccer, analyzing 80 matches across four age groups: U15, U17, U19, and NB1 (adults). Team-level data were examined to assess how maturation affects the integration of physical and technical demands. Physical metrics included total distance (TD) and total high-intensity distance (THID), while TP metrics involved actions such as pressing, tackling, and ball possession. Pearson’s correlations and general linear models (GLMs) were used to identify associations between PP and TPs across age categories. The results showed age-related trends in performance integration. U19 and NB1 players demonstrated moderate-to-strong correlations between PP and TP variables, with TD and THID positively associated with pressing and tackling, indicating increasing synergy with age. U17 players showed emerging integration, while U15 players exhibited no significant correlations, suggesting developmental variability. Limitations include reliance on team-level correlational data and the absence of individual physiological or cognitive assessments. These findings suggest that training approaches should be tailored to players’ developmental stages, with increasing emphasis on combining physical conditioning and tactical understanding as players mature.

## 1. Introduction

Soccer requires the advanced and simultaneous development of technical, tactical, physical, and psychosocial skills. The interdependence of these domains becomes particularly relevant as youth players progress through age-based categories, where developmental milestones strongly influence performance outcomes [1,2,3]. In addition to age, tactical maturity and biological growth play crucial roles in shaping players’ physical and technical proficiencies, which are instrumental in driving success in competitive matches [4]. Notably, physical, technical, and tactical performances do not develop in isolation but interact within the dynamic context of match play, where external factors, such as match venue, fixture density, and league standing, further shape performance [5,6,7].

In adult populations, tactical maturity and positional demands often dictate physical performance parameters, with certain roles and formations requiring higher-intensity outputs and specific skill sets [8,9]. For example, players in wide positions, such as midfielders and fullbacks, tend to cover greater distances at high intensities compared to central players, owing to their broader field coverage requirements [8,10]. However, these adult-based insights may not fully apply to youth players, whose performance metrics are influenced by developmental stages that affect physiological and cognitive readiness [11,12]. Recent studies in soccer emphasize the importance of a progression-based approach to player development that incorporates tactical understanding and technical skills alongside physical training to support youth players’ holistic growth [13,14].

Despite substantial research on adult soccer performance [15,16,17,18], there remains a notable gap in studies focusing on youth players, where physical, tactical, and technical demands are shaped by age-related biological and cognitive development. Maturation biology suggests that different physical abilities peak at distinct stages, with physiological functions often reaching their maximum before cognitive capacities can be fully harnessed and applied to match scenarios [11,12]. This discrepancy underscores the need to understand how age affects key performance indicators, such as total distance (TD) and total high-intensity distance (THID), in youth players, which could provide valuable insights for optimizing training strategies that align with their evolving physiological and tactical capacities.

This study is grounded in the conceptual framework that views physical and tactical development as mutually reinforcing processes. As youth players mature, both biologically and cognitively, their ability to integrate physical capacities—such as high-intensity running—with complex tactical behaviors like pressing and positional play is expected to improve. This interplay forms the basis of our analytical approach across age categories. Accordingly, the present study aimed to examine how the interaction between physical performance and technical–tactical actions evolves with age in youth soccer. We hypothesized that older age groups (U19 and NB1) would demonstrate stronger correlations between physical and technical–tactical metrics compared to younger groups (U15 and U17), reflecting their higher levels of biological and tactical maturity.

## 2. Materials and Methods

### 2.1. Sample

The study sample comprised a total of 80 soccer matches, with corresponding team-level data recorded across four age groups: under-15 (U15), under-17 (U17), under-19 (U19), and adults (NB1). The average ages of the players at the time of the matches were 14.2 ± 0.9 years for U15, 16.5 ± 0.7 years for U17, 18.5 ± 1.2 years for U19, and 27.5 ± 2.2 years for the NB1 adult team. This segmentation allowed for an age-specific analysis to observe performance trends across youth categories. Specifically, data were collected from 18 matches for the U15 age group, 16 matches for the U17 group, 26 matches for the U19 group, and 20 matches for the NB1 team. Importantly, all matches involved teams competing at the highest level of the Hungarian national championship within their respective age categories. Therefore, the findings should be interpreted in the context of elite national-level competition, which supports external validity within similarly high-performance settings. Although this structure allowed us to identify age-related trends at the team level, it inherently limits insights into player-level variability or positional effects. To control for differences in team formation, all matches analyzed employed a 4-3-3 tactical formation, a structure documented to influence player movement and field coverage uniquely [9]. However, we acknowledge that selection bias may be present due to the dependence on data availability through the club and the InStat system. The inclusion criteria may also favor matches with higher tracking fidelity or availability, which could impact the representativeness of the sample. We mitigated this by including a balanced number of matches per age group, as far as the available data allowed. All data collection adhered to the principles of the Declaration of Helsinki to ensure player and team confidentiality, with ethical approval obtained from the local university ethics board (approval number: MTSE-OKE_KEB/10/2023).

### 2.2. Procedures

Data collection on physical performance was conducted using an advanced athlete monitoring system equipped with 10 Hz GPS units and 100 Hz triaxial accelerometers (Vector S7, Catapult Sports, Melbourne, Australia). These GPS units are positioned in each player’s upper back, just above the shoulder blades, which provides a stable location to capture movement data accurately across all game phases. The reliability and validity of this technology for capturing velocity-based data have been well-documented in prior studies, with results indicating consistent accuracy across a range of sports contexts [19,20,21]. GPS technology has significantly advanced the study of sports performance, providing critical insights into players’ movement patterns and intensity levels during matches. Malone et al. (2017) [22] underscore the importance of adhering to standardized GPS data collection protocols to reduce inter-unit variability, which is particularly relevant in youth soccer, where monitoring consistency ensures accurate analysis. In this study, each player used the same GPS unit throughout the data collection period, minimizing variability and enhancing data reliability [21].

Technical performance data were collected using a multicamera, semi-automated optical tracking system (InStat Fitness, Instat Limited, Limerick, Ireland), which captures player movements through stationary cameras at a 25 Hz sampling frequency. This system has received FIFA Quality certification for Electronic and Performance Tracking Systems (EPTSs), ensuring high reliability and accuracy in monitoring technical metrics like passing accuracy, dribbling success, and shooting [23]. Physical parameters assessed included total distance (TD) and total high-intensity [>19.8 km h^−1^] distance (THID), while technical–tactical parameters (TPs) included a variety of team actions such as pressing, tackling, and ball possession. The technical–tactical parameters and associated definitions are presented in Table 1

Data normality was confirmed using the Kolmogorov–Smirnov test (all K-S *p* > 0.05), and homogeneity was assessed with Levene’s test. The statistical analyses were conducted in multiple phases. As an initial step, we applied a general linear model analysis, using TPs and the age groups as the predictors and total distance and total high-intensity distance as the outcome variables. We calculated the model’s coefficients of determination (R^2^) and determined the relative contribution of TPs within the model. Finally, Pearson’s correlation coefficients were calculated to establish associations between TPs and PP, with correlation strength classified as follows: *r* < 0.35 indicates a weak correlation, *r* = 0.35 to 0.67 represents a moderate correlation, *r* = 0.68 to 0.9 is considered a strong correlation, and *r* > 0.9 a very high correlation [24]. GraphPad Prism (Version 10.2.2) was used for all statistical analyses, with the significance level set at *p* < 0.05.

## 3. Results

The descriptive values for each parameters are presented in Table S1.

The general linear model indicated that the predictors explained 75.5% of the variance in total distance (TD), underscoring the combined influence of ball possession and age group. For total high-intensity distance (THID), 66.7% of the variance was accounted for, with only the age group emerging as a significant predictor (Table 2).

Table 3 presents the detailed associations between technical–tactical parameters (TPs) and physical performance (PP). Within the NB1 cohort, TD demonstrated moderate positive correlations with passes, accurate passes, total actions, and successful actions, each accounting for approximately 20–24% of the shared variance. Additionally, THID exhibited a moderate correlation with shots on target. In the U19 group, TD was moderately and negatively associated with ball possession, whereas positive moderate correlations were observed with team pressing and high pressing, each explaining approximately 16% of the variance. THID further showed moderate associations with successful low pressing, tackles, and successful tackles in this age group. Among U17 players, TD correlated moderately with successful team pressing, tackles, and successful tackles, collectively explaining around 31% of the shared variance, while THID did not demonstrate significant associations with any TP metrics. No significant correlations were identified in the U15 group, where most technical–tactical parameters accounted for less than 10% of the common variance. Importantly, no correlation exceeded *r* = 0.68, indicating an absence of strong or very strong associations as per the defined thresholds.

## 4. Discussion

The findings of this study highlight distinct age-related differences in the relationship between physical (PP) and technical–tactical performance (TP) parameters across youth soccer age groups, underscoring the substantial role of biological and tactical maturity in influencing these associations. As prior research indicates, the age at which players develop their tactical and physical skills is critical to their performance trajectory, with mature players typically demonstrating a higher integration of physical and technical abilities compared to their younger counterparts [11]. This study’s data on the U19 and NB1 age groups align with these findings, showing stronger correlations between physical and technical metrics, such as total distance (TD) and team pressing, as well as high-intensity running (THID) and successful low pressing actions. These results mirror those of Aquino et al. (2020) [8], who emphasized that tactical roles, such as pressing, significantly shape physical demands in mature players, leading to more cohesive performance profiles in older age groups.

The lack of a significant correlation in the U15 group should be interpreted with caution. While it may suggest developmental differences in tactical understanding and physical capabilities [11,12], this absence could also reflect greater variability, limited sample sensitivity, or noise inherent to the early developmental stages. Without direct physiological or cognitive assessments, we cannot conclusively attribute the lack of association to underdeveloped tactical or physical cohesion. Unlike older players, younger youth players are still refining basic motor skills and tactical awareness, which are critical for effectively integrating technical skills with high-intensity physical actions [25]. This developmental limitation supports the notion that tactical and physical abilities in youth soccer should be fostered progressively, starting with foundational skills in younger age groups before introducing more complex tactical and physical demands [5,13]. Furthermore, Brindescu et al. (2021) emphasize that advanced pressing tactics, which require considerable cognitive and physical maturity, should be gradually introduced, as these tactics demand high levels of decision-making and endurance, especially in youth transitioning to higher age categories [26].

Additionally, our findings affirm previous research that links ball possession strategies with reduced physical load, a concept well-documented in studies on adult and elite soccer populations. Lorenzo-Martinez et al. (2021) [17] found that teams with higher ball possession cover less total distance and high-intensity running, indicating that ball control can lower physical demands. A similar trend was observed in the U19 age group, where a moderate negative correlation between ball possession and TD was noted. However, without control for context-specific factors such as opponent strength, possession duration, or formation dynamics, this finding should be interpreted cautiously. Although previous research suggests that ball control may contribute to lower physical demands [27,28], further investigation is necessary to confirm this relationship in youth settings. Such insights suggest that coaches may need to adjust training protocols to balance physical conditioning with tactical maturity, focusing on possession drills that foster technical skills without overloading players physically in later developmental stages. The lack of association between ball possession and PP in the NB1 group reinforces previous trivial findings [15,16] and could be the result of other contextual factors like as match venue, fixture density, and league standing [5,6,7].

Our results also resonate with research on repeated-sprint ability in youth, which shows improvement in high-intensity physical metrics as players age [4]. The positive correlation between THID and successful pressing and tackling actions observed in the U19 group indicates that players in this stage have achieved a level of maturity where they can maintain high physical outputs during technically demanding actions. The importance of repeated-sprint ability has been previously linked to successful tactical execution in professional soccer, where high-intensity efforts are often required for effective pressing and counter-pressing actions [29]. Therefore, the integration of high-intensity physical performance with technical metrics in the older age groups in our study not only highlights maturation but also underscores the need for age-specific conditioning programs that build endurance in tandem with tactical skills.

A critical implication of these findings is the necessity for age-adapted training approaches that account for players’ maturity stages. Youth soccer development programs frequently focus on skill acquisition in isolation, yet our results suggest that technical skills should be gradually combined with physical conditioning to foster a cohesive performance framework as players age. Studies advocate for developmental models that align with athletes’ cognitive and physical milestones, which can be instrumental in designing age-appropriate training regimes that cater to tactical, technical, and physical dimensions [18,30]. A progression-based model in youth soccer training, where intensity and complexity are incrementally increased, could thus help bridge the maturity gap, ensuring younger players transition smoothly to more advanced tactical roles as they age [14].

These insights suggest that, with appropriate caution, coaches may benefit from prioritizing a holistic training approach that balances cognitive, tactical, and physical development. Given the moderate correlations observed, training recommendations should be made conservatively and ideally be supplemented by individual assessments of players’ physiological and tactical readiness. Gonçalves et al. (2021) [13] found that such integrated training approaches promote greater tactical flexibility and decision-making, which are invaluable as youth players progress to higher levels. In practical terms, training programs for U15 players should focus on skill refinement and foundational motor tasks, while U17 and U19 players can benefit from more intense, tactical-focused drills that incorporate pressing, tackling, and counter-attacks. Aligning training with age-appropriate cognitive and physical goals can also mitigate injury risks, allowing players to develop endurance and technical precision safely [8].

The current study has several limitations that should be acknowledged. First, the sample was limited to 80 soccer matches across four age categories (U15, U17, U19, NB1), and analyses were performed at the team level rather than the individual player level. This approach may obscure within-team variability and limits conclusions regarding individual differences. Second, data were collected under a single tactical formation (4-3-3), which, while controlling for formation-related variability [9], may reduce the generalizability of findings to other tactical setups. Third, we did not control for players’ biological maturation status or playing positions, both of which are known to influence physical and tactical performance metrics in youth soccer. These factors could have introduced additional variability into the associations observed. Fourth, our analysis was correlational in nature, which precludes any causal inferences regarding the relationship between physical and technical–tactical parameters. Fifth, although match location, fixture density, and league standing are known to impact performance metrics [5,6,7], and we mention them, we also lacked other contextual control variables (e.g., opponent quality, possession duration, match dynamics), limiting the specificity of tactical–physical associations. However, a recent study has shown that situational factors have minimal influence on PP in elite adult matches [31]; thus, their impact on the results of the current study is unknown. Lastly, the smaller sample size, particularly in the U15 and U17 groups, may have resulted in lower statistical power, possibly obscuring true but weaker associations within these cohorts. Future research should explore these variables within larger datasets, incorporating additional performance metrics (e.g., high-intensity sprints, tactical success rates) across varied formations and situational contexts to provide a more comprehensive understanding of youth player development.

## 5. Conclusions

This study provides preliminary insights into how biological and tactical maturity may influence the relationship between physical and technical parameters in youth soccer. The findings suggest that while younger players (U15) may exhibit greater variability in tactical and physical integration, older groups (U17 and U19) show emerging patterns of performance synergy. These trends align with previous research on youth athletic development, supporting the notion that a gradual integration of physical and technical training, tailored to players’ age and developmental stage, could be beneficial [4,11]. However, given the correlational nature of the study, the team-level design, and the absence of causal modeling, the results should be interpreted cautiously. Any practical recommendations for training adaptations should be considered exploratory rather than prescriptive. Coaches and practitioners may find these findings useful as a general guide, but individualized assessments remain essential when designing training programs. Future research, particularly longitudinal and player-centered studies, is needed to confirm these preliminary observations and refine development strategies. Moreover, advancements in GPS-based tracking technologies offer promising avenues for capturing nuanced developmental trajectories across different stages of youth soccer. A progression-based, holistic approach remains a promising framework, but further empirical validation is necessary to optimize performance outcomes and safely guide athletes through critical phases of their development.

## Figures and Tables

**Table 1:** The technical-tactical parameters and their definitions.

Parameters	Definitions
**Accurate passes**	Total number of accurate passes
**Ball Possession**	Active possession time for the whole match
Crosses	Number of long passes performed by a player from an offensive zone (last 40 m of pitch between the short side of the penalty area and the lateral side of the field) and directly of the penalty area
Crosses accurate	Total number of accurate crosses
Entrances to the opponent’s box	Number of entries into the opponent’s penalty area
Goal Chances	Number of created goal chances, finished with a shot or without a shot
High pressing	Total number of collective attempts to force the opponents to lose the ball or to stop the development of an attack on the opponent’s half of the pitch
High pressing successful	Number of successfully performed high pressings
Key passes	Passes to a partner who is in a goal scoring position (one-on one situation, empty net, etc.). And passes to partner that “cuts off” the whole defensive line of the opponent’s team (3 or more player) in the attacking phase
Key passes accurate	Total number of accurate key passes
Low pressing	Total number of collective attempts to force the opponents to lose the ball or to stop the development of an attack on one’s own half of the pitch
Low pressing successful	Number of successfully performed low pressings
Number of successful pressing actions (in units)	A successfully executed joint possession strategy based on a successful territory constriction, at the end of which the team regains possession of the ball
Number of team pressing actions (number)	Joint ball retrieval based on area narrowing. This closes off the playability of the ball and narrows down the scope for individual solutions
Passes	Total number of passes
Shots	Total number of shots to score
Shots on target	Number of shots within a goal
Successful actions	Total number of successful actions
Tackles	Active action of a player who tries to tackle the ball from the player possessing it
Tackles successful	Total number of successful tackles
Team possession percentage (%)	The possession time for each team is divided by the active possession time for the whole match
Team pressing	A combination based on area reduction ball retrieval. This closes the playability of the ball and narrows the individual solutions
Team pressing successful	A successfully implemented area narrowing a joint ball retrieval strategy, at the end of which the team is again possession of the ball
Total actions	Total number of all types of actions (including passes, crosses, set pieces passes, tackles, challenges, shots, etc.)

**Table 2:** General linear model for total distance and total high-intensity distance.

	Total Distance				Total High-Intensity Distance			
	F	p	ηp^2^	Power	F	p	ηp^2^	Power
Intercept	168.06	0.00	0.76	1.00	12.47	0.00	0.19	0.93
Age group	17.65	0.00	0.50	1.00	15.54	0.00	0.46	1.00
Goal Chances	0.20	0.66	0.00	0.07	0.08	0.78	0.00	0.06
Shots	0.12	0.73	0.00	0.06	0.02	0.90	0.00	0.05
Shots on target	0.00	1.00	0.00	0.05	0.38	0.54	0.01	0.09
Passes	3.29	0.08	0.06	0.43	0.95	0.33	0.02	0.16
Accurate passes	2.45	0.12	0.04	0.34	1.84	0.18	0.03	0.27
Key passes	0.21	0.65	0.00	0.07	0.26	0.61	0.00	0.08
Key passes accurate	0.25	0.62	0.00	0.08	0.24	0.62	0.00	0.08
Crosses	0.32	0.57	0.01	0.09	0.94	0.34	0.02	0.16
Crosses accurate	0.01	0.91	0.00	0.05	0.91	0.34	0.02	0.16
Team pressing	0.75	0.39	0.01	0.14	0.01	0.94	0.00	0.05
Team pressing successful	0.36	0.55	0.01	0.09	0.50	0.48	0.01	0.11
High pressing	0.05	0.83	0.00	0.06	0.03	0.94	0.00	0.06
High pressing successful	0.28	0.60	0.01	0.08	0.43	0.52	0.01	0.10
Low pressing	0.46	0.50	0.01	0.10	0.01	0.94	0.00	0.05
Low pressing successful	0.74	0.39	0.01	0.13	0.23	0.63	0.00	0.08
Ball Possession	4.98	0.03	0.08	0.59	1.03	0.32	0.02	0.17
Tackles	1.33	0.25	0.02	0.20	2.26	0.14	0.04	0.31
Tackles successful	0.97	0.33	0.02	0.16	2.70	0.11	0.05	0.37
Entrances to the opponent’s box	0.83	0.37	0.02	0.15	0.00	1.00	0.00	0.05
Total actions	0.30	0.59	0.01	0.08	0.10	0.76	0.00	0.06
Successful actions	0.62	0.44	0.01	0.12	0.58	0.45	0.01	0.12
	R^2^ = 0.755				R^2^ = 0.667			

ηp^2^ Partial Eta Squared.

**Table 3:** Associations between technical–tactical performance and physical performance.(data are given as r (p)).

	Total Distance				Total High-Intensity Distance			
	NB1	U19	U17	U15	NB1	U19	U17	U15
Ball Possession	0.19 (0.426) (low)	**−0.40 (0.041) (moderate)**	−0.10 (0.709) (low)	−0.10 (0.697) (low)	−0.03 (0.913) (low)	−0.34 (0.088) (low)	0.03 (0.915) (low)	−0.10 (0.686) (low)
Goal Chances	−0.10 (0.669) (low)	0.25 (0.216) (low)	0.36 (0.173) (moderate)	0.00 (0.997) (low)	0.32 (0.166) (low)	0.22 (0.276) (low)	0.42 (0.107) (moderate)	0.12 (0.648) (low)
Shots	−0.15 (0.541) (low)	0.44 (0.255) (moderate)	0.25 (0.360) (low)	0.10 (0.697) (low)	0.07 (0.769) (low)	0.22 (0.273) (low)	0.41 (0.110) (moderate)	0.28 (0.265) (low)
Shots on target	0.18 (0.460) (low)	0.30 (0.140) (low)	0.36 (0.165) (low)	0.03 (0.919) (low)	**0.46 (0.044) (moderate)**	0.26 (0.198) (low)	0.46 (0.073) (moderate)	0.16 (0.523) (low)
Passes	**0.45 (0.045) (moderate)**	−0.18 (0.378) (low)	0.03 (0.924) (low)	0.01 (0.980) (low)	0.08 (0.730) (low)	−0.34 (0.091) (low)	−0.05 (0.864) (low)	−0.20 (0.421) (low)
Accurate passes	**0.45 (0.046) (moderate)**	−0.18 (0.371) (low)	−0.02 (0.941) (low)	0.00 (0.998) (low)	0.10 (0.685) (low)	−0.36 (0.068) (moderate)	−0.05 (0.854) (low)	−0.19 (0.453) (low)
Key passes	−0.24 (0.298) (low)	0.26 (0.197) (low)	0.15 (0.567) (low)	−0.16 (0.517) (low)	0.14 (0.548) (low)	0.20 (0.325) (low)	0.19 (0.474) (low)	−0.13 (0.602) (low)
Key passes accurate	−0.12 (0.601) (low)	0.31 (0.117) (low)	0.19 (0.482) (low)	−0.13 (0.614) (low)	0.39 (0.089) (moderate)	0.17 (0.399) (low)	0.21 (0.442) (low)	−0.21 (0.424) (low)
Crosses	0.09 (0.706) (low)	−0.15 (0.478) (low)	0.21 (0.446) (low)	0.03 (0.899) (low)	−0.02 (0.943) (low)	−0.11 (0.585) (low)	−0.13 (0.629) (low)	0.09 (0.729) (low)
Crosses accurate	0.20 (0.406) (low)	−0.08 (0.716) (low)	0.14 (0.604) (low)	−0.02 (0.934) (low)	0.17 (0.473) (low)	−0.02 (0.907) (low)	0.21 (0.433) (low)	0.20 (0.434) (low)
Team pressing	0.13 (0.573) (low)	**0.41 (0.040) (moderate)**	0.44 (0.086) (moderate)	0.30 (0.222) (low)	−0.07 (0.758) (low)	0.13 (0.515) (low)	0.14 (0.603) (low)	0.27 (0.278) (low)
Team pressing successful	−0.14 (0.570) (low)	0.07 (0.725) (low)	**0.57 (0.021) (moderate)**	0.35 (0.155) (moderate)	−0.33 (0.158) (low)	−0.10 (0.615) (low)	0.31 (0.249) (low)	0.30 (0.221) (low)
High pressing	−0.06 (0.809) (low)	**0.41 (0.035) (moderate)**	0.33 (0.219) (low)	0.29 (0.244) (low)	−0.16 (0.507) (low)	0.28 (0.166) (low)	0.00 (0.997) (low)	0.15 (0.551) (low)
High pressing successful	−0.10 (0.692) (low)	0.31 (0.125) (low)	0.50 (0.050) (moderate)	0.30 (0.222) (low)	−0.22 (0.356) (low)	0.21 (0.309) (low)	0.17 (0.525) (low)	0.22 (0.384) (low)
Low pressing	0.27 (0.252) (low)	0.20 (0.324) (low)	0.29 (0.273) (low)	0.11 (0.678) (low)	0.11 (0.632) (low)	−0.18 (0.388) (low)	0.23 (0.402) (low)	0.23 (0.369) (low)
Low pressing successful	0.00 (0.998) (low)	−0.36 (0.080) (moderate)	0.24 (0.361) (low)	0.09 (0.713) (low)	−0.20 (0.399) (low)	**−0.50 (0.011) (moderate)**	0.29 (0.282) (low)	0.15 (0.549) (low)
Tackles	0.22 (0.356) (low)	0.22 (0.269) (low)	**0.54 (0.030) (moderate)**	−0.14 (0.589) (low)	0.13 (0.585) (low)	**0.52 (0.006) (moderate)**	−0.02 (0.946) (low)	−0.14 (0.570) (low)
Tackles successful	0.03 (0.888) (low)	0.18 (0.391) (low)	**0.57 (0.022) (moderate)**	−0.31 (0.216) (low)	−0.01 (0.959) (low)	**0.41 (0.035) (moderate)**	−0.08 (0.771) (low)	−0.32 (0.193) (low)
Entrances to the opponent’s box	−0.16 (0.501) (low)	0.03 (0.883) (low)	0.27 (0.310) (low)	−0.06 (0.808) (low)	0.14 (0.570) (low)	−0.17 (0.413) (low)	0.43 (0.094) (moderate)	0.14 (0.583) (low)
Total actions	**0.49 (0.028) (moderate)**	−0.02 (0.922) (low)	0.16 (0.544) (low)	−0.08 (0.751) (low)	0.14 (0.566) (low)	−0.12 (0.549) (low)	0.07 (0.794) (low)	−0.17 (0.494) (low)
Successful actions	**0.48 (0.032) (moderate)**	−0.12 (0.572) (low)	0.12 (0.654) (low)	−0.02 (0.932) (low)	0.15 (0.528) (low)	−0.27 (0.183) (low)	0.05 (0.844) (low)	−0.16 (0.538) (low)

Bold text represents statistical significance *p* < 0.05.

## Data Availability

The data are available upon reasonable request to the corresponding author. The data are not publicly available due to privacy reasons.

## Supplementary Materials

The following supporting information can be downloaded at https://www.mdpi.com/article/10.3390/sports13060162/s1: Table S1: Mean, standard deviation and confidence interval for each variable analyzed, presented per group.
