# Supplementary material for: Age-Related Effects of Physical Performance on Technical and Tactical Outcomes in Youth Soccer
Source: Sports (Basel). 2025 May 27;13(6):162. doi: 10.3390/sports13060162 (PMC12196848; doi:10.3390/sports13060162)
Supplement: Supplementary file 1 [file sports-13-00162-s001.zip › Table S1.pdf]

	NB1				U19				U17				U15		
	**Mean±SD**	**Lower limit (CI95 %)**	**Upper limit (CI95 %)**		**Mean±SD**	**Lower limit (CI95 %)**	**Upper limit (CI95 %)**		**Mean±SD**	**Lower limit (CI95 %)**	**Upper limit (CI95 %)**		**Mean±SD**	**Lower limit (CI95 %)**	**Upper limit (CI95 %)**
Total Distance	106836.0±4948.0	104520.0	109152.0		103191±5231	101078.0	105304.0		104529±3653	102582.0	106475.0		90995±4758	88629.0	93361.0
Total High-Intensity Distance	6819.0±953.7	6372.0	7265.0		6519±985	6121.0	6916.0		6897±664	6543.0	7251.0		4523±897	4077.0	4969.0
Goal Chances	7.6±3.3	6.08	9.12		5.5±3.1	4.263	6.737		8.1±3.9	6	10		7.2±3.3	5.6	8.9
Shots	13.7±4.7	11.5	15.8		12.73±3.6	11.29	14.17		13±4.3	11	15		13±6.9	10	17

Shots on target	5.95±2.6	4.75	7.15		5.077±2.1	4.246	5.908		5.5±3.1	3.9	7.1		6.9±3.7	5	8.7
Passes	580±104	531	629		426.6±86.8	391.6	461.7		416±86	370	462		373±66	340	406
Accurate passes	495±97.7	449	541		342.3±86.1	307.6	377.1		332±86	286	378		281±67	248	315
Key passes	9.8±5.4	7.26	12.3		5.962±2.6	4.93	6.993		7.9±3.4	6.1	9.8		6.8±3.6	5.1	8.6
Key passes accurate	4.75±2.6	3.56	5.94		2.731±1.5	2.11	3.352		4.4±2	3.3	5.4		4.1±2.5	2.8	5.3

Crosses	13.4±7.5	9.83	16.9		12±4.6	10.12	13.88		8.6±3.9	6.5	11		8±4.7	5.7	10
Crosses accurate	2.95±2.1	1.98	3.92		3.308±1.8	2.596	4.019		2.6±1.8	1.6	3.5		2.7±1.6	1.9	3.5
Team pressing	16.7±4.1	14.8	18.6		21.15±8.9	17.57	24.74		23±6.9	19	26		21±5.9	18	24
Team pressing successful	7.8±3.2	6.32	9.28		11.15±4.9	9.16	13.15		12±4.6	9.2	14		11±3.4	9.3	13
High pressing	8.15±4.0	6.26	10		11.65±6.7	8.929	13.38		12±5.5	9.5	15		9.6±4.7	7.3	12

High pressing successful	4.47±2.6	3.23	5.72		6.885±3.9	5.3	8.469		6.8±4	4.7	9		5.9±3.3	4.3	7.6
Low pressing	8.55±2.9	7.18	9.92		9.5±4.0	7.891	11.11		10±4.3	8.1	13		11±3.9	9.1	13
Low pressing successful	3.55±1.8	2.73	4.37		4.44±2.3	3.494	5.386		4.9±2.5	3.5	6.2		5.1±2.1	4	6.1
Ball Possession	59.7±8.1%	56.1%	63.2%		53.9±9.1 %	50.0%	57.3%		50.1±7.5 %	46.0%	55.0%		51.0±6.5 %	46.0%	56.0%
Tackles	26.6±5.8	23.9	29.3		45.58±14.7	39.6	51.53		49±14	41	56		49±13	43	55

Tackles successful	14.4±3.8	12.6	16.2		26.08±5.9	23.7	28.45		28±9	24	33		27±7.4	23	31
Entrances to the opponent's box	18.3±5.8	15.6	21		16.69±3.8	15.14	18.25		17±5.5	14	20		15±6.4	12	18
Total actions	878±111	826	930		798.8±74.6	768.7	828.9		807±106	750	863		746±77	708	784
Successful actions	704±102	657	752		592.5±79.0	560.6	624.4		598±96	547	649		535±76	497	573
